# Efficacy of Different Volumes of 0.2% Ropivacaine in Suprainguinal Fascia Iliaca Compartment Block for Multimodal Analgesia in Lower Limb Surgery

**DOI:** 10.7759/cureus.46894

**Published:** 2023-10-12

**Authors:** Jessica Sekhon, Richa Jain, Kamya Bansal, Neeru Luthra, Mirley R Singh, Bindu Kumari

**Affiliations:** 1 Anaesthesiology, Dayanand Medical College and Hospital, Ludhiana, IND

**Keywords:** local anesthetics, femur fracture, volume, analgesia, ropivacaine, ultrasound

## Abstract

Background

Ultrasound-guided (USG) suprainguinal fascia iliaca (SIFI) block is being used widely for post-operative analgesia in patients undergoing hip and femur surgeries. However, the optimal volume of local anesthetic required for SIFI block is not well defined. Thus, we compared different volumes of 0.2% ropivacaine in SIFI for post-operative pain relief in lower limb surgeries.

Material and methods

A total of 90 patients undergoing hip and femur surgeries were randomly allocated into three groups: A, B, and C, who received USG SIFI block with 20 mL, 30 mL, and 40 mL of 0.2% ropivacaine, respectively. Intravenous tramadol was used as rescue analgesia when the numeric rating scale (NRS) score exceeded 3. Time to first request of rescue analgesic was the primary outcome. NRS scores in the first 24 hours post-operatively, total amount of tramadol consumption in 24 hours, and patient satisfaction with pain management were secondary outcomes.

Results

The time to first request to rescue analgesic was significantly longer in group B and group C as compared to group A. NRS scores were significantly reduced in group B and group C than group A in the 24-hour post-operative period. Median 24-hour tramadol consumption was significantly less in group C as compared to group A and group B. Patient satisfaction with pain management was better with group B and group C as compared to group A.

Conclusion

In comparison to 20 mL of 0.2% ropivacaine, 30 mL and 40 mL of 0.2% ropivacaine in SIFI compartment block are more efficacious in reducing post-operative pain after hip and lower limb surgeries.

## Introduction

Surgery for femur and hip fractures is known to be associated with moderate-to-severe post-operative pain for the first 24-48 hours, which may adversely affect early mobilization and physiotherapy, eventually affecting rehabilitation of the patient. This pain is primarily due to extensive bony and soft tissue damage and instrumentation. Adequate pain control is an important part of post-operative care for enhanced recovery after surgery as severe pain may lead to an increased stress response and unanticipated hemodynamic changes, which can then trigger major cardiovascular and cerebrovascular complications such as cerebral hemorrhage and myocardial infarction. Thus, early and aggressive application of safe and effective analgesic modalities is essential to improve the outcomes in these patients, especially for older adults with multiple underlying diseases [1,2].

Previous studies have shown that nerve blocks effectually reduce the pain from femur fractures and provide rapid-onset local analgesia, which is more effective than conventional analgesia [3]. Peripheral nerve blocks such as femoral nerve (FN) block, Winnie's 3- in-1 FN block, pericapsular nerve group block, and fascia iliaca compartment block (FICB) have been used to provide post-operative analgesia with opioid-sparing effects [4-6]. FICB is more often used for analgesia in patients with hip fractures [7,8]. FICB is a regional anesthesia technique in which a local anesthetic is deposited in the potential fascial plane below the fascia iliaca and above the iliopsoas muscle containing the FN, the obturator nerve (ON), and the lateral femoral cutaneous nerve (LFCN). It can be approached either by the conventional infrainguinal method or the modified proximal suprainguinal method. The infrainguinal approach requires a larger volume of local anesthetic agent than the suprainuinal approach to block the LFCN and articular branches of FN, which are placed proximal to the inguinal ligament [9,10]. As a result, in order to block these nerves, the drug must move superiorly from the thigh, necessitating a larger volume.

Recently, ultrasound-guided suprainguinal approach has been proposed as a novel method for performing FICB. As opposed to the classical approach, the suprainguinal approach directs the puncture needle cephalad, allowing for easier spread and improved analgesic effects from only a small amount of medication [11,12]. However, there are only a few studies [13,14] comparing different effective volumes of local anesthetic used for the block for achieving successful post-operative analgesia in hip and femur surgeries. Therefore, we herein performed a randomized, controlled, double-blind clinical trial to compare the efficacy of different volumes of 0.2% ropivacaine (20 mL, 30 mL, and 40 mL) administered under ultrasound guidance in the suprainguinal FICB for post-operative pain relief in patients undergoing above knee lower limb surgeries under subarachnoid block.

## Materials and methods

Trial design and registration

This is a double-blind, randomized, prospective interventional study that was registered at the Clinical Trials Registry - India (CTRI) (vide reference number CTRI/2021/09/036144) and approved by the Institutional Ethics Committee.

Participants

A total of 90 patients, aged >18 years, of American Society of Anesthesiologists (ASA) grades I, II, and III, undergoing above knee lower limb surgeries under subarachnoid block were recruited for the study. The above knee lower limb surgeries included hip hemiarthroplasty and proximal femur fixations. The procedure was conducted in accordance with the Helsinki Declaration 2013. Exclusion criteria were patient refusal, contraindications to the suprainguinal FICB (coagulopathies, infection at injection site, allergy to local anesthetic), chronic opioid abuse, and neuropathies.

Randomization and blinding

Patients were divided into three study groups (A, B, and C with 30 patients each) by computer-generated randomization codes contained in sealed, sequentially numbered envelopes. The patients as well as the observer involved in the study were kept unaware of the group allocation. The allocation envelope was opened by an anesthesiologist who was administering the block to the patient. A blinded observer assessed pre- and post-operative pain scores. These codes were decoded at the end of the study period. Patients in groups A, B, and C received 20 mL, 30 mL, and 40 mL of 0.2% ropivacaine, respectively, in suprainguinal fascia iliaca (SIFI). A written informed consent was obtained from each patient.

Procedure

On the operating table, patients were positioned in the supine position and ASA monitoring was instituted in the form of 5-lead electrocardiography, non-invasive blood pressure, temperature, and pulse oximetry. Baseline pain score at rest using the numeric rating scale (NRS) was recorded prior to the administration of block. NRS score was determined by asking the patient to rate the pain from 0 to 10, with 0 being the least pain and 10 being the maximum pain. With the patient in the supine position, the anterior superior iliac spine (ASIS) was palpated. An anesthesiologist stood on the side of block, and a linear high-frequency probe (6-14 MHz, FUJIFILM Sonosite Edge II, FUJIFILM Sonosite, Inc., Bothell, WA) was placed in the inguinal crease to identify the femoral artery in the short axis. From the femoral artery, the transducer was translated laterally to identify the sartorius muscle. The sartorius was traced cephalad to its insertion on ASIS. Using this technique, the hypoechoic shadow of the ASIS was easily identiﬁable just cephalad to the insertion of the sartorius. Medial to the shadow of the ASIS lied the iliacus muscle. With the ASIS and iliacus identiﬁed, the medial end of the transducer was rotated to point at the umbilicus, which was the ﬁnal transducer position. With the transducer in the ﬁnal position, sonographic anatomy was identiﬁed, from superﬁcial to deep, consisting of subcutaneous fat, the internal oblique muscle, the transverse abdominus muscle, the fascia iliaca overlying the iliacus muscle, and the iliacus muscle itself. A 21-gauge, 100-cm stimuplex ultra 360 (B Braun Medical, Melsungen, Germany) needle was introduced 1 cm cephalad to the inguinal ligament. Using an in-plane approach, the needle tip was positioned beneath the fascia iliaca, and hydrodissection was used to separate the fascia iliaca from the iliacus muscle. The needle was then further advanced in this space in a cranial and slightly dorsal direction. An injection was considered successful if spread of the local anesthetic is observed cranial to the point where the iliacus muscle passes under the abdominal muscles. If adequate spread was not observed, the needle was repositioned. A proper injection was ensured by the separation of fascia iliaca by the local anesthetic in the mediolateral direction. A successful block was defined as a visible deposition of ropivacaine in the fascia iliaca plane as viewed on ultrasound. After 15 minutes of block or when the NRS score was <4, the patient was administered a subarachnoid block in the sitting position with 2.5 mL of 0.5% bupivacaine heavy and 25 mcg of fentanyl via a 26G Quincke needle. Post-operatively, injection paracetamol 1 g intravenously (IV) was given six hourly to all patients. Injection tramadol 50 mg IV was used as a rescue analgesic whenever the NRS score was ≥4, with a maximum of 150 mg within 24 hours; in case the NRS score was still >4, then another rescue analgesic, injection fentanyl 1 mcg/kg IV, was administered as a slow bolus. NRS for pain was recorded post-operatively, immediately on shifting to the post-anesthesia care unit, and then every hour for the first 4 hours and then two hourly for the next 8 hours and then four hourly till 24 hours post-operatively. Post-operative observations were recorded by another anesthesiologist.

Outcomes

Primary outcome was the time to first requirement of rescue analgesic, which was defined as the time when the NRS scale score was ≥4 for the first time post-surgery, with time 0 being the time of initiation of block. NRS pain scores, total requirement of rescue analgesics (tramadol and fentanyl) in the first 24 hours after surgery, and patient satisfaction with the pain management were recorded as secondary outcomes. Patient satisfaction score was measured by asking the patient to rate their satisfaction on a 5-point Likert scale (very satisfied, satisfied, neither satisfied nor dissatisfied, dissatisfied, very dissatisfied) at 24 hours after surgery.

Statistical analysis

Data were defined in terms of range: mean ± standard deviation (±SD), median, frequencies (number of cases), and relative frequencies (percentages) as appropriate. Quantitative variables (age, BMI, weight, time to first request of rescue analgesic, total 24 hours of rescue analgesic consumption) between the study groups were compared using Student’s t-test and Mann-Whitney’s U test for independent samples for parametric and non-parametric data, respectively. For comparing categorical data (sex, ASA grade, type of surgery, patient satisfaction), chi-square (χ2) test was used, and Fischer’s exact test was used when the expected frequency would be less than 5. A probability value (p-value) of less than 0.05 was considered statistically significant. All statistical calculations were conducted using Statistical Package for the Social Science (SPSS) Version 21 (IBM Corp., Armonk, NY).

Sample size estimation

The sample size was estimated from the results previous studies [12,15,16] using the time to first request of analgesia as the parameter, which is the primary outcome of our study. Our sample size came out to be 30 subjects per group at a power of 0.89, an effect size of 0.38, 10% chance of error, α = 0.05, β = 0.20, and confidence interval of 95%.

## Results

**Figure 1:**
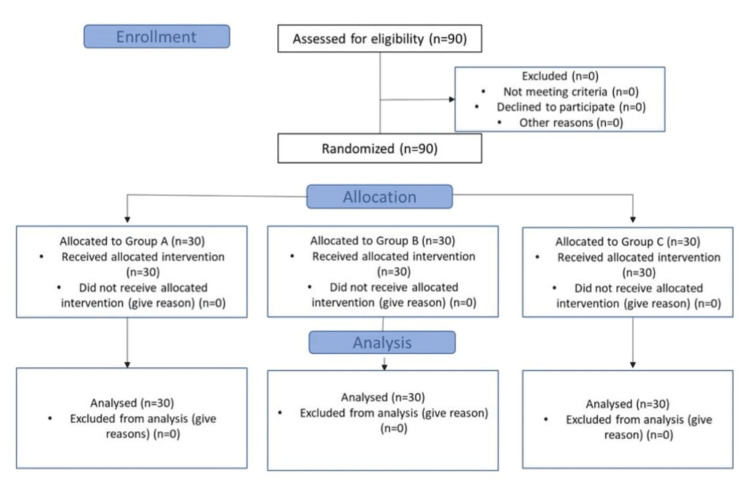
CONSORT flow diagram of the study n = number of patients; CONSORT, Consolidated Standards of Reporting Trials

A total of 90 patients were enrolled in the study, and all of them completed the study (Figure 1). They were randomly allocated to either of the three groups (A, B, C) with 30 patients each using computer-generated random numbers. All of them received the interventions, and there were no dropouts.

All three groups were statistically comparable with respect to the demographic data (Table 1).

**Table 1:** Demographic profile of the three groups *Values expressed as mean (standard deviation) n, number of cases; ASA, American Society of Anesthesiologists

Variable		Group A	Group B	Group C	Chi-square value/F	P-value
Age (years)*		65.57 (10.21)	61.63 (20.79)	58.03 (19.47)	1.396	0.253
Sex, n	F	16	14	12	1.071	0.585
	M	14	16	18		
Weight (kg)*		70.60 (13.84)	72.07 (8.71)	71.87 (12.61)	0.134	0.875
BMI(kg/m^2^)*		25.38 (4.00)	26.72 (3.52)	24.95 (3.17)	2.008	0.140
ASA grade, n	I	1	3	5	4.111	0.391
	II	10	8	11		
	III	19	19	14		
Type of surgery, n	Femur	17	21	23	28.656	0.095
	Hip	13	9	7		

**Figure 2:**
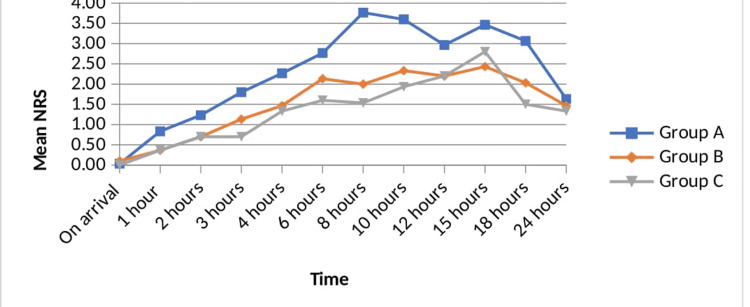
Comparative trends in mean NRS scores at rest post-operatively. NRS, numeric rating scale

Figure 2 shows the trends in mean NRS scores in all three groups at arrival of patients to the post-anesthesia care unit till 24 hours post-operatively. Statistically, significantly reduced NRS scores were observed at rest in group B and group C than group A at all hours except the 24th hour (p<0.05).

Time to first rescue analgesic or the mean duration of analgesia was 577.83 + 138.95 minutes in group A, 728.26 + 313.96 minutes in group B, and 937.35 + 31.33 minutes in group C. The difference was statistically significant among all three groups (p=0.023) (Table 2).

**Table 2:** Comparison of outcomes observed in the three groups. *Values expressed as mean (standard deviation); **median (IQR)

	Group A	Group B	Group C	Chi-square	P-value	P-value		
						Group A vs B	Group A vs C	Group B vs C
Time to request of first analgesia (minutes)*	577.83 (138.95)	728.26 (313.96)	937.35 (31.33)	17.200	0.001	0.025	0.000	0.005
Cumulative analgesic consumption (tramadol; mg)**	131.67 (33.43)	91.30 (51.46)	50.00 (0.00)	27.405	0.001	0.001	0.000	0.002

Seven patients in group B and 13 patients in group C did not require any rescue analgesic for 24 hours, with NRS scores ranging from 0 to 3. Median values of total rescue analgesic consumption and patient satisfaction score among the three groups are shown in Table 2. Median 24-hour tramadol consumption was significantly lower in group C as compared to group A and group B (p<0.05). Patient satisfaction scores (Table 3) were better in group B and group C as compared to group A (p=0.001). No patient required fentanyl for rescue analgesia.

**Table 3:** Patient satisfaction score in three groups.

		Group A	Group B	Group C	Chi-square	P-value
Patient satisfaction score (no. of cases)	Very satisfied	0	7	29	109.455	0.001
	Satisfied	4	21	0		
	Neither satisfied nor dissatisfied	26	2	1		
	Dissatisfied	-	-	-		

## Discussion

SIFI compartment block can efficiently reduce pain associated with above knee lower limb surgeries as it has the potential to create a sensory block of the FN, ON, and LFCN [1]. Thus, we included hip hemiarthroplasties and proximal femur fixations, which have a similar level of surgical dissection and incision line well above the knee.

We chose to use 0.2% ropivacaine due to its significantly low neurotoxic and cardiotoxic potential. In addition, it results in a lower incidence of motor blockade secondary to motor sensory differentiation, thereby further supporting early mobilization and safety in the post-operative period [17].

In our study, all three groups were statistically comparable with respect to demographic profile, type of surgery, duration of surgery, and anesthesia. Hence, the comparable confounders did not have the ability to bias our results.

In our study, the time to request of first rescue analgesic was significantly prolonged in group B and group C than in group A. None of the studies compared the duration of analgesia while giving different volumes of local anesthetic in SIFI block.

Post-operative pain score, in terms of NRS, was observed to be significantly decreased in group B and group C as compared to A at predominantly all measurement time points of the study in the post-operative period. Chen et al. observed significantly lower visual analog scale (VAS) scores at 6 and 12 hours after procedure with 30 mL of 0.2% ropivacaine in SIFI block in comparison to infrainguinal FICB in 50 patients undergoing hip surgeries [1]. Azizoğlu et al. demonstrated significantly lower pain scores at rest till 6 hours and pain scores with movement for up to 24 hours post-operatively with 40 mL of 0.25% bupivacaine in USG SIFI in comparison to patient-controlled analgesia with morphine [2]. Though we found no studies comparing different volumes of drug required for SIFI block, the results of studies evaluating the effective volume for the same were corresponding to ours. A randomized controlled trial was conducted by Yamada et al. [13] to determine the 50% effective volume (EV50) and 95% effective volume (EV95) of 0.25% ropivacaine in ultrasound-guided suprainguinal FICB. EV50 and EV95 of 0.25% ropivacaine for ultrasound-guided suprainguinal FICB calculated with logistic regression analysis were 15.01 mL (95% confidence interval: 6.53- 22.99) and 26.99 mL (95% confidence interval: 20.54-84.09), respectively. These results are in concordance with the findings of our study, where 30 mL of 0.2% ropivacaine in ultrasound-guided FICB provided significantly prolonged time to first request of rescue analgesia, reduced total requirement of IV tramadol, and better patient satisfaction scores, as compared to lower doses of ropivacaine.

The total requirement of opioid analgesics (tramadol) in the first 24 hours after surgery was significantly lower in group C as compared to group B and group A in our study. Desmet et al. hypothesized that a higher dose of ropivacaine (0.5%) is essential for successful analgesia in suprainguinal FICB [12]. They used 40 mL of 0.5% ropivacaine in the test group and no block in the control group. Mean morphine consumption at 24 hours post-operatively was observed to be reduced in group FICB compared to the control group: 10.25 mg versus 19.0 mg (p=0.004). We observed similar results in our study, where we safely administered higher volume (40 mL) of 0.2% ropivaciane in suprainguinal FICB and observed significantly prolonged pain-free periods, reduced total IV tramadol requirement in 24 hour post-operative period, and better patient satisfaction with pain management.

Quality of block assessed by patient satisfaction score at the end of 24 hours was also observed to be significantly better with group B and group C as compared to group A in our study. We found no literature on patient satisfaction among different volumes of SIFI block.

These findings imply superior analgesic efficacy of 30 mL and 40 mL over 20 mL of 0.2% ropivacaine in suprainguinal FICB. A preliminary cadaveric study conducted by Vermeylen observed the spread of different volumes in a single-injection suprainguinal FICB in cadavers via computed tomography (CT) scan as well as dissection by a blinded anatomist [14]. They observed that 20 mL of volume spread to the FN and LFCN but to the ON. Combined CT scan and dissection findings showed that 40 mL of injection adequately spread to all three nerves, i.e., FN, LFCN, and ON. In addition, 40 mL of injection also showed better spread to the psoas muscle and ON as compared to 30 mL. These findings are in accordance to the observations of our study where 40 mL of 0.2% ropivacaine in suprainguinal FICB, as compared to 30 mL and 20 mL, showed prolonged pain-free periods, significantly reduced requirement of IV tramadol, and better patient satisfaction in the 24-hour post-operative period.

A double-blind comparative study was conducted by Helayel et al. to calculate the effective volume of 0.5% ropivacaine and 0.5% bupivacaine in 50% (EV50%), 95% (EV95), and 99% (EV99) of the cases to achieve FICB [18]. The volume of anesthetic capable of producing effective nervous anesthesia in 50% of the cases was observed to be 28.79 mL (95% CI: 26.31-31.5 mL) for ropivacaine and 29.56 mL (95% CI: 25.22-34.64 mL) for bupivacaine (p=0.62). The effective volumes of ropivacaine capable of effectively nervous anesthesia in 50%, 95%, and 99% of the cases were estimated by prohibits regression as 28.8 mL (95% CI: 27.2-30.4), 34.3 mL (95% CI: 32.5-37.3), and 36.6 mL (95% CI: 34.3-40.5), respectively. The corresponding volumes of bupivacaine were 29.5 mL (95% CI: 28.1-31.1), 36.1 mL (95% CI: 33.5-38.1), and 37.3 mL (95% CI: 35.1-41.3) (p>0.05). In our study, we observed similar findings, where 30 mL and 40 mL of 0.2% ropivacaine provided better 24 hour post-operative pain relief in terms of prolonged duration of analgesia, reduced IV tramadol requirement, and better patient satisfaction.

The patients in our study did not exhibit respiratory depression, pruritus, nausea, and vomiting, probably due to the opioid-sparing effects of the suprainguinal FICB. In our study, we gave single-shot FICB over continuous FICB as it is simpler and easy to administer with comparable opioid-sparing effect. Continuous FICB requires insertion of perineural catheters, which are expensive and require maintenance.

There were a few limitations in our study. Firstly, all patients received spinal anesthesia, which could be a confounding factor resulting in a variable effect on post-operative analgesic requirement. For this purpose, patients enrolled in this study were of similar demographic characteristics, and a standard dose of 12.5 mg of bupivacaine with 25 micrograms of fentanyl was used. Secondly, block was given after spinal anesthesia; thus, the analgesic efficacy of block could not be assessed. But despite this limitation, one major advantage our study design offered was that the blocks were administered using ultrasound guidance, resulting in better success rates in terms of analgesic efficacy. Thirdly, the post-operative pain was assessed at rest only; dynamic pain was not assessed. Also, we used the Likert scale for assessment of patient satisfaction score. However, it is multifactorial and thus a poor assessment tool, which is another limitation of the study.

## Conclusions

Ninety patients undergoing hip hemiarthroplasties and proximal femur fixation under spinal anesthesia were enrolled in our study. They all received successful SIFI block for post-operative pain. From the results of our study, we conclude that 30 mL and 40 mL of 0.2% ropivacaine in suprainguinal FICB are equally efficacious in significantly reducing pain in the post-operative period after hip and lower limb surgeries in terms of NRS scores, time to first rescue analgesic, and patient satisfaction scores. Also, these volumes were significantly more effective for providing post-operative analgesia as compared to 20 mL.
